# Hypoxia Activates the K-Ras Proto-Oncogene to Stimulate Angiogenesis and Inhibit Apoptosis in Colon Cancer Cells

**DOI:** 10.1371/journal.pone.0010966

**Published:** 2010-06-04

**Authors:** Min Zeng, Hirotoshi Kikuchi, Maria S. Pino, Daniel C. Chung

**Affiliations:** Gastrointestinal Unit, Department of Medicine, Massachusetts General Hospital, Harvard Medical School, Boston, Massachusetts, United States of America; Health Canada, Canada

## Abstract

The *KRAS* proto-oncogene plays a key role in the development of many human tumors and is commonly activated by somatic mutation or signaling through specific growth factor receptors. However, the interaction between the micro-environment and K-ras activity has not been defined. Hypoxia invariably develops as tumors outgrow their supply of oxygen. A series of well-orchestrated cellular adaptations occur that stimulate angiogenesis and enhance survival of the tumor in hypoxic conditions. Our previous studies demonstrated that mutant *KRAS* alleles can interact with hypoxia to induce vascular endothelial growth factor (VEGF) in colon cancer. We sought to determine whether similar hypoxic responses are also present in tumors without a *KRAS* mutation. Hypoxia consistently increased the levels of activated, GTP-bound K-ras in colon cancer cell lines with a wild-type *KRAS* gene, and this depended upon the activation of c-Src. Inhibition of c-Src by PP2 treatment or siRNA knockdown blocked the hypoxic activation of K-ras. This activation of K-ras did not depend upon EGFR and resulted in the phosphorylation of Akt and induction of VEGF expression. In addition, activation of K-ras significantly blocked apoptosis in hypoxic conditions. These studies reveal a unique adaptive mechanism in hypoxia that activates K-ras signaling in the absence of a mutant *KRAS* oncogene.

## Introduction

The development of tissue hypoxia is characteristically observed as malignant tumors rapidly increase in size. Such hypoxic conditions exert selective pressure on cancer cells, and the ability of tumor cells to survive in a hypoxic microenvironment has been associated with a poor prognosis and resistance to therapy [1]. One of the most critical and best characterized responses to hypoxia is the induction of vascular endothelial growth factor (VEGF), and hypoxia-inducible factor-1 (HIF-1) is a well-established mediator of this process. However, previous studies have demonstrated that HIF-independent mechanisms can also induce VEGF in hypoxia, and oncogenic K-ras plays a key role in this process [2], [3], [4].

K-ras is a small GTPase that cycles between inactive guanosine diphosphate (GDP)-bound and active guanosine triphosphate (GTP)-bound conformations (Ras-GDP and Ras-GTP, respectively) [5]. It serves as a signal switch molecule that couples receptor activation by specific growth factors with downstream effector pathways including the Ras-MEK-ERK and phosphatidylinositol 3-kinase (PI3K)-Akt cascades that control multiple cellular responses. Oncogenic mutations in *KRAS* impair the ability of K-Ras to hydrolyze bound GTP in a growth factor-independent manner, and constitutive signaling through these effector pathways results. The tumor microenvironment can have a profound influence on cellular behavior, and hypoxia has been shown to interact with many oncogenic signaling pathways. In particular, hypoxic activation of the PI3K-Akt, MEK-ERK, NF-κB, and hypoxia-inducible factor (HIF) signaling pathways has been described [6], [7]. Although K-ras is a central regulator in all these pathways, it is unknown whether K-ras itself is specifically activated by hypoxia. Previous studies have demonstrated a strong synergistic interaction between hypoxia and mutant K-ras in the regulation of multiple target genes including vascular endothelial growth factor (VEGF), IL-8, and osteopontin [2], [8], [9]. However, fewer than 50% of colon tumors harbor *KRAS* mutations, and the relationship between Ras signaling and hypoxia in tumors with wild-type *KRAS* remains undefined.

We sought to determine the role of K-ras in the hypoxic micro-environment in colon cancer, a tumor type that frequently harbors *KRAS* mutations. Wild-type K-ras was strongly activated by hypoxia, and c-Src was necessary for this hypoxic activation of K-ras. This resulted in the phosphorylation of Akt and induction of VEGF expression. In addition, hypoxic activation of wild-type K-ras blocked apoptosis. Collectively, these findings highlight a new mechanism for the hypoxic activation of oncogenic survival pathways in the absence of an oncogenic mutation.

## Results

### Hypoxic activation of K-Ras in colon cancer cells

**Figure 1:**
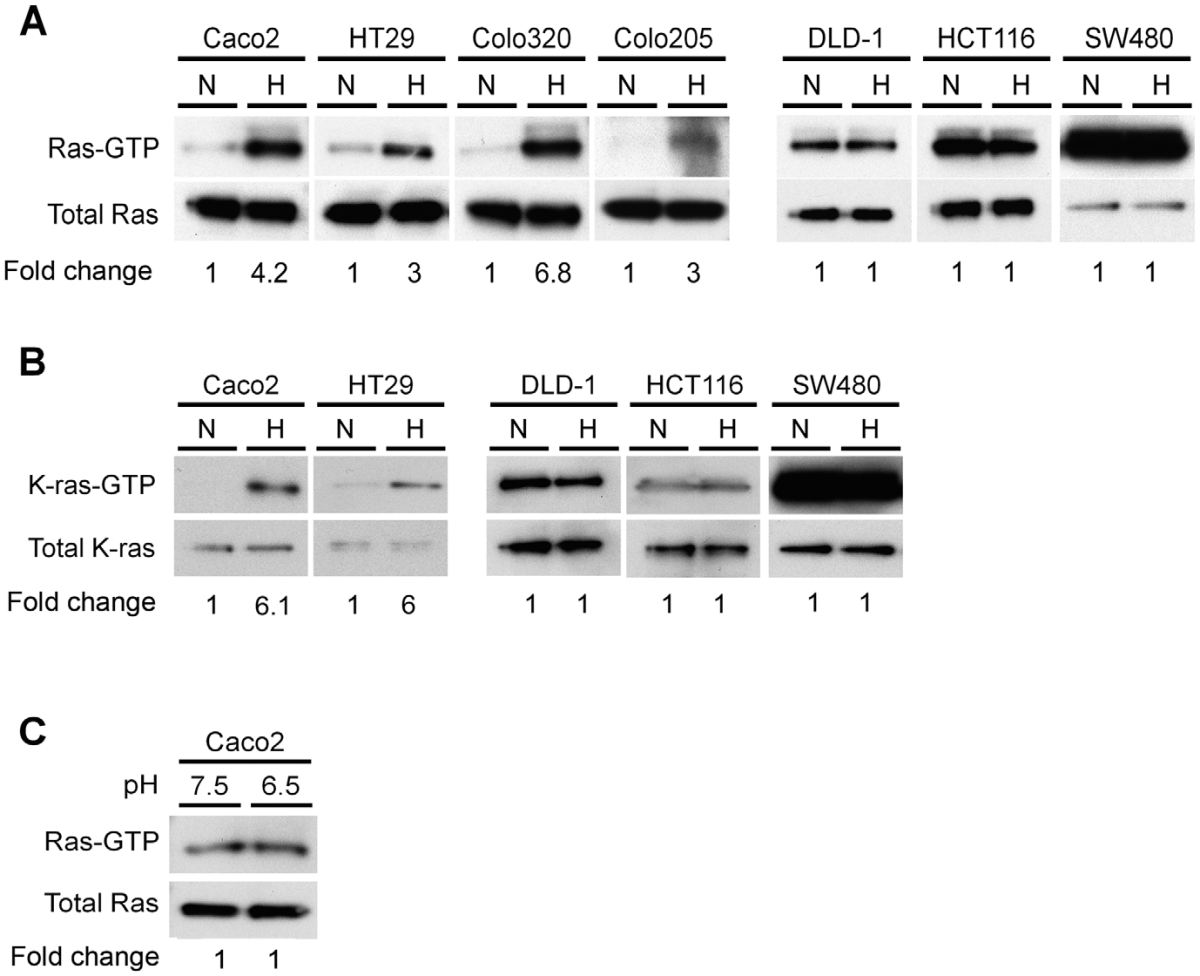
Hypoxia activates Ras in colon cancer cell lines with a wild-type *KRAS*. The levels of GTP-bound Ras (**A**) and GTP-bound K-ras (**B**) were evaluated in wild-type (left panel) and mutant (right panel) *KRAS* cell lines grown either in normoxic (N) or hypoxic (H) conditions for 4 hours. Two milligrams of cell extracts were used for a Ras activation assay, followed by Western blotting with the indicated antibodies. Densitometry values are expressed as fold change compared with control values normalized to 1. **C**, Caco2 cells were cultured in DMEM with pH adjusted to 7.5 or 6.5 for 4 hours. Cell lysates were used for a Ras activation assay followed by Western blotting with a Ras-GTP specific antibody. Densitometry values are expressed as fold change compared with control values normalized to 1.

To determine whether hypoxia activates Ras, we measured GTP-bound Ras in normoxic and hypoxic conditions in a panel of colon cancer cell lines. Levels of GTP-bound Ras were barely detectable in Caco2, HT29, Colo320DM and Colo205 cells in normoxic conditions (Fig. 1A). All of these lines have a wild-type *KRAS* gene. However, when these cells were incubated in hypoxic conditions (1% O_2_), there was a dramatic increase (from 3 to 6.8 fold) in the levels of activated Ras (Fig. 1A). In contrast, the basal activities of Ras in DLD1, HCT116 (both heterozygous for the *KRAS^D13^* mutation), and SW480 (homozygous for the *KRAS^V12^* mutation) cells in normoxia were high, and there was no subsequent increase in hypoxia (Fig. 1A, right panel). SW480 cells had the strongest levels of Ras activation in normoxic conditions. Hypoxia is thus a strong activator of Ras in colon cancer cells, but this induction was observed only in those cells with a wild-type *KRAS* gene.

To verify that the K-ras isoform was activated by hypoxia, a K-ras specific antibody was utilized. As illustrated in Figure 1B, levels of GTP bound-K-ras were induced by hypoxia in colon cancer cells with wild-type *KRAS* (Caco2, HT29) whereas there were no changes in cell lines with a mutant *KRAS* oncogene (DLD1, HCT116, SW480). A role for N-ras in the regulation of apoptosis has been suggested in colon cancer, but an N-ras specific antibody failed to detect GTP-bound-N-ras in Caco2, DLD1, and HCT116 cell lines in either normoxic or hypoxic conditions (Fig. S1) [10].

Tumor hypoxia is often accompanied by a switch to anaerobic glycolysis and a lower pH, so we investigated whether the hypoxic regulation of Ras activity was mediated through changes in pH. Incubation of Caco2 cells in acidic conditions (pH 6.5) for 4 hours did not increase the activity of Ras, suggesting that changes in pH do not regulate levels of GTP-bound Ras (Fig. 1C).

### Upregulation of Ras activity by hypoxia requires c-Src

**Figure 2:**
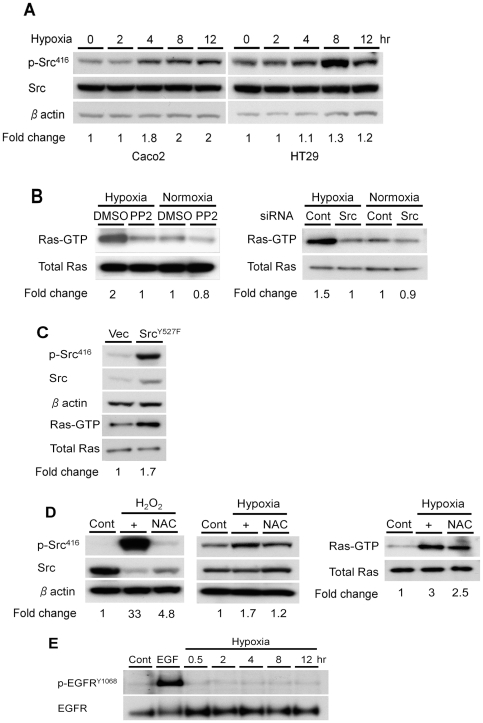
Activation of Ras by hypoxia is dependent on c-Src activation. **A**, Caco2 and HT29 cells were incubated in hypoxic conditions for the indicated times, and Western blotting for phospho-Src^416^ and total Src was performed. β-actin confirmed equal loading. Densitometry values are expressed as fold change compared with control values normalized to 1. **B**, Caco2 cells were pretreated with the Src inhibitor PP2 (20 µM) or DMSO for 1 hour (left panel), or transiently transfected with c-Src specific siRNA constructs or a non targeting control (right panel), before incubation in hypoxic or normoxic conditions for 4 hours. Activated Ras was pulled down and SDS-PAGE was performed using a Ras-GTP specific antibody. Total Ras confirmed equal loading. Densitometry values are expressed as fold change compared with control values normalized to 1. **C**, Lysates of Caco2pEVX and Caco2SrcY527F cells were immunostained for p-Src^416^ and total Src and also used for a Ras activation assay. Densitometry values for Ras-GTP are expressed as fold change compared with control values normalized to 1. **D**, **left panel**, Control cells and cells pretreated with NAC (20 mM) for 20 minutes were exposed for 10 minutes to H_2_O_2_ (5 mM). Lysates were then immunoblotted for p-Src^416^ and total Src. β-actin confirmed equal loading. **Middle and right panels**, Control Caco2 cells and cells pretreated with NAC (20 mM) for 1 hour were incubated in hypoxia for 4 hours. Western blotting for phospho-Src^416^ (middle panel) and a Ras activating assay (right panel) were then performed. Densitometry values are expressed as fold change compared with control values normalized to 1. **E**, Lysates of control Caco2 cells and cells incubated in hypoxia for the indicated times were subjected to Western blotting to detect p-EGFR (Tyr^1068^) and total EGFR protein levels. Treatment with EGF (100 ng/mL) served as a positive control.

c-Src lies upstream of K-ras, and we next sought to explore whether c-Src is also activated by hypoxia. A time-dependent activation of c-Src, as measured by phosphorylation at Tyr^416^, was observed in both Caco2 and HT29 cells after incubation in hypoxic conditions (Fig. 2A). To determine if c-Src activity contributed to the activation of Ras by hypoxia, we pretreated Caco2 cells with PP2, a potent and selective Src tyrosine kinase inhibitor. A 50% reduction in the hypoxic induction of Ras was detected in Caco2 cells, while there was no effect on the levels of total Ras (Fig. 2B, left panel). This effect was dose-dependent, with a maximal effect seen at 20 µM (data not shown).

To more directly assess the role of c-Src, we knocked down endogenous c-Src in Caco2 cells via RNA interference. Knock-down of c-Src led to a 33% reduction in the hypoxic activation of GTP-bound Ras (Fig. 2B, right panel). Furthermore, Caco2 cells expressing a constitutively active c-Src vector (SrcY527F) exhibited a 70% increase in the level of GTP-bound Ras (Fig. 2C) when compared with empty vector (pEVX). Western blotting verified the K-ras isoform was activated by expression of Src (Fig. S2).

Reactive oxygen species (ROS) generated by hypoxia have previously been shown to activate Src. Exogenous administration of H_2_O_2_ (5 mM) potently induced phosphorylation of Src at Tyr^416^ in Caco2 cells (Fig. 2D, left panel). This effect was significantly reduced by NAC (20 mM), an antioxidant that inhibits ROS. We then sought to determine whether NAC could block the hypoxic activation of endogenous Src and Ras. NAC treatment reduced the induction of p-Src^416^ in hypoxia by approximately 30% with a concomitant 17% reduction of GTP-bound Ras (Fig. 2D). These findings suggest that generation of ROS may partially contribute to the hypoxic activation of c-Src in colon cancer cells.

Recent reports have suggested that hypoxia can increase levels of EGFR and that phosphorylation of EGFR may be an intermediate step in the signaling from Src to Ras [11], [12]. To determine whether EGFR may play such a role, we measured total EGFR and phospho-EGFR levels in Caco2 cells incubated in hypoxia. There was a small increase in total EGFR in hypoxia. However, there was no increase in EGFR phosphorylation at Tyr^1068^ (Fig. 2E). Overall, the data suggest that up-regulation of c-Src activity by hypoxia is mediated in part through ROS but that activation of K-Ras by c-Src in hypoxia does not depend upon EGFR.

### Activation of Akt by hypoxia is downstream of c-Src and K-ras

**Figure 3:**
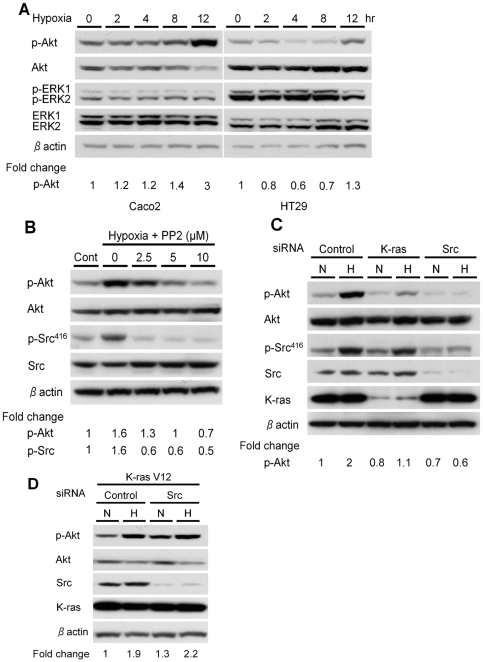
Activation of Akt by hypoxia is downstream of c-Src and K-ras. **A**, Protein extracts from Caco2 and HT29 cells grown in normoxic or hypoxic conditions for the indicated times were subjected to immunoblotting for phospho-Akt and phospho-ERK1/2. The blots were then stripped and reprobed with antibodies against total Akt and total ERK. β-actin was used as a loading control. Densitometry values for p-Akt are expressed as fold change compared with control values normalized to 1. **B**, Caco2 cells were incubated for 1 hour with PP2 at the concentrations indicated, before transfer to normoxic or hypoxic conditions for 12 hours. Western blotting was performed to determine the levels of phospho-Akt, total Akt, phospho-Src^416^, and total Src. β-actin was used as a loading control. Densitometry values are expressed as fold change compared with control values normalized to 1. **C**, Caco2 cells were transfected with control siRNA, K-ras siRNA or c-Src siRNA oligos (each at 20 nM) before exposure to normoxic (N) or hypoxic (H) conditions for 12 hours. Immunoblotting with the indicated antibodies was then performed. Densitometry values for p-Akt are expressed as fold change compared with control values normalized to 1. **D**, Caco2 cells stably overexpressing K-rasV12 (Caco2pCSGWK-ras V12) were transfected with c-Src siRNA oligos. Forty-eight hours later, cells were incubated in hypoxia or normoxia for 12 hours and Western blotting with the indicated antibodies was then performed. Densitometry values for p-AKT are expressed as fold change compared with control values normalized to 1.

We next sought to determine which downstream effector pathways were activated by c-Src and K-ras in hypoxia. Exposure to hypoxic conditions induced phosphorylation of Akt at Ser^473^ in both Caco2 and HT29 cells, while no activation of ERK was detected under the same conditions (Fig. 3A). Akt was activated in a time-dependent manner and reached a maximum level at 12 hours. To determine whether Src was involved in the hypoxic activation of Akt, we pretreated Caco2 cells with the specific Src inhibitor PP2 for 12 hours before exposure to hypoxia. As shown in Figure 3B, the hypoxic activation of Akt was reduced by PP2 treatment in a dose-dependent manner. Consistent with these findings, knock down of c-Src also completely blocked the hypoxic activation of Akt when compared to cells transfected with a control siRNA (Fig. 3C).

Because Akt is known to be downstream of K-ras, we tested whether Akt is also a specific target of K-ras under hypoxic conditions by utilizing K-ras siRNA oligos. Silencing of K-ras reduced the levels of Akt in hypoxia when compared to cells transfected with control siRNA (Fig. 3C). These data indicate that hypoxic activation of Akt is mediated through both Src and K-ras. No changes in phospho-Src^416^ and total Src protein levels were observed when K-ras was silenced (Fig. 3C), indicating that Src is indeed upstream of K-ras in this hypoxic signaling pathway.

To independently verify that K-ras was downstream of Src in this hypoxia-induced phosphorylation of Akt, we performed experiments in a Caco2 cell line stably expressing the mutant oncogene *KRAS^V12^* (Caco2/pCSGWK-rasV12). The hypoxic induction of p-Akt was not blocked by silencing of c-Src with siRNA (Fig. 3D). These findings indicate that c-Src functions upstream of K-ras in the hypoxic activation of Akt.

### K-ras and Src mediate resistance to apoptosis in hypoxia

**Figure 4:**
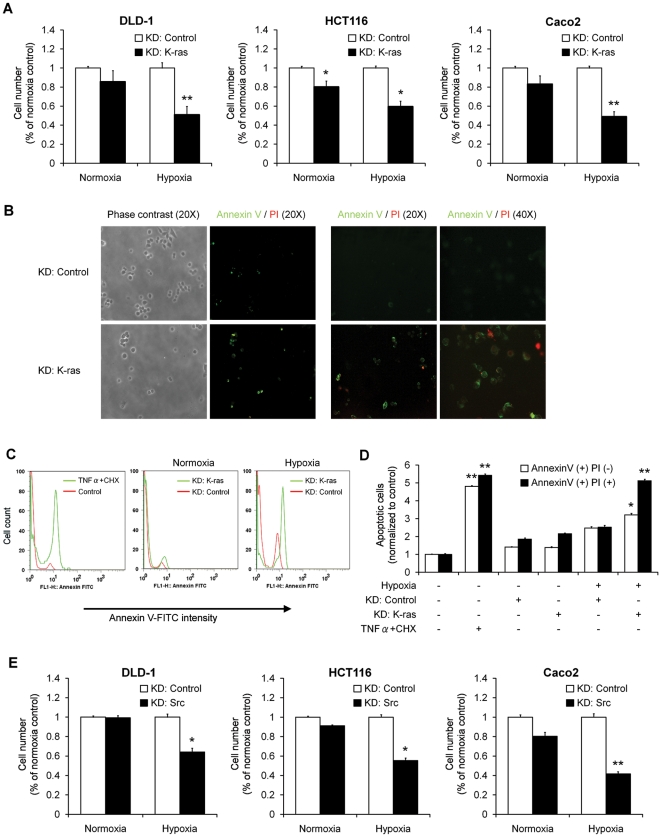
K-ras and Src enhance the survival of colon cancer cells in hypoxia. **A**, DLD1, HCT116 and Caco2 cells were transfected with control siRNA or K-ras siRNA, and then incubated in hypoxia for 48 hours. Cell numbers were determined using a hemacytometer after staining with trypan blue and the results are expressed as percentages of viable cells compared with siRNA control transfected cells in normoxia. The data are from three independent experiments and shown as mean ± SD. *, P<0.05; **, P<0.01. **B**, Colo320DM cells grown on sterile coverslips were transfected with siRNA control oligos (shown in top rows) or siRNA oligos to K-ras (shown in bottom rows) for 24 hours, followed by incubation in hypoxia for 48 hours. Early apoptotic (FITC+PI-) and late apoptotic/necrotic cells (FITC+PI+) were detected. Left panels: 20x phase contrast and 20x fluorescence green and red channel merged images. Right panels: 20x and 40x fluorescence green and red channel merged images. **C**, Caco2 cells were transfected with K-ras siRNA or control siRNA, and then incubated in normoxia or hypoxia for 48 hours. Cell death was determined by FACS as described in Materials and Methods. **D**, Early apoptotic (Annexin V+PI-) and late apoptotic/necrotic (Annexin V+PI+) cells were determined by FACS analysis. Mean ± SD of three independent experiments is shown. *, P<0.05. **, P<0.01. **E**, DLD1, HCT116 and Caco2 cells were transfected with control siRNA or c-Src siRNA oligos (20 nM) for 24 hours and then exposed to hypoxia for 48 hours. Cells excluding trypan blue were counted and results are expressed as percentage of viable cells compared with siRNA control transfected cells in normoxia. Mean ± SD of three independent experiments is shown. *, P<0.05. **, P<0.01.

Akt plays a pivotal role in regulating apoptosis and cell cycle progression. Since Akt is a downstream target of K-ras in this hypoxia-triggered intracellular signaling pathway, we sought to determine whether silencing of K-ras would affect cell survival. Knock-down of K-ras decreased cell viability even in normoxic conditions by 20% in HCT116 cells, but there were no significant changes in DLD1 or Caco2 cells. In contrast, K-ras knockdown in hypoxic conditions reduced cell counts to a much greater extent: a 40% reduction in HCT116 (*P*<0.05) and 50% reductions in DLD1 and Caco2 cells (*P*<0.01) were observed (Fig. 4A). Silencing of K-ras in Caco2 cells resulted in a survival rate in hypoxia comparable to DLD1 and HCT116 cells that harbor a mutant *KRAS* gene, suggesting that the wild-type K-ras protein also plays an important role in the adaptive mechanism in hypoxia.

We then labeled cells with FITC-conjugated annexin V and propidium iodide (PI) to measure rates of apoptosis. Another wild-type *KRAS* cell line, Colo320DM, was studied because its growth pattern as single cells permits visualization of apoptotic membrane changes more clearly. Silencing of K-ras resulted in alterations in cell morphology, including blebbing, shrinkage, and nuclear fragmentation, as well as reduced attachment to the tissue culture dish and increased floating. Silencing of K-ras also increased the number of Annexin V positive cells compared with control siRNA (Fig. 4B). To quantify the anti-apoptotic effects of K-ras in hypoxia, we analyzed apoptotic cell populations by flow cytometry (FACS). Caco2 cells transfected with either a non targeting control or K-ras siRNA oligos were incubated in normoxia or hypoxia for 48 hours. Cells without any treatment served as a negative control, and cells exposed to UV light for 10 minutes and cultured with TNF-α and Cycloheximide (CHX) served as a positive control (Fig. 4C, left panel). A histogram of Annexin V-FITC positive cells revealed that knock-down of K-ras in normoxia did not significantly alter rates of apoptosis. However, under hypoxic conditions, loss of K-ras significantly increased rates of cell death from 22% to 57% when compared with control siRNA treatment (Fig. 4C, middle and right panel). We further analyzed the apoptotic cells as two subpopulations: early apoptotic cells (Annexin V+, PI-) and late apoptotic/necrotic cells (Annexin V+, PI+). Only when K-ras was knocked-down under hypoxic conditions was a significant induction of apoptosis observed by FACS analysis; there was a 1.3-fold increase in the number of early apoptotic cells (*P*<0.05) and 2.0-fold increase in the number of late apoptotic cells (*P*<0.01) when compared to control siRNA in hypoxia (Fig. 4D). These studies indicate that K-ras can inhibit apoptosis in hypoxia.

Our data suggested that Src may also regulate survival pathways that block apoptosis in hypoxia. We defined the relationship between Src and cell survival directly by counting viable cells that excluded trypan blue. Knock-down of Src in DLD1 and HCT116 cells did not alter cell survival in normoxia (1% and 9% reductions in cell counts, respectively), whereas in hypoxia, cell counts decreased by 36% and 45%, respectively (Fig. 4E). Similarly, knockdown of c-Src in Caco2 cells reduced cell counts by 20% in normoxia compared to a more significant 58% reduction in hypoxia. Therefore, the activation of K-ras or Src under hypoxic conditions enhances the survival of colon cancer cells.

### Hypoxic regulation of VEGF by K-ras

**Figure 5:**
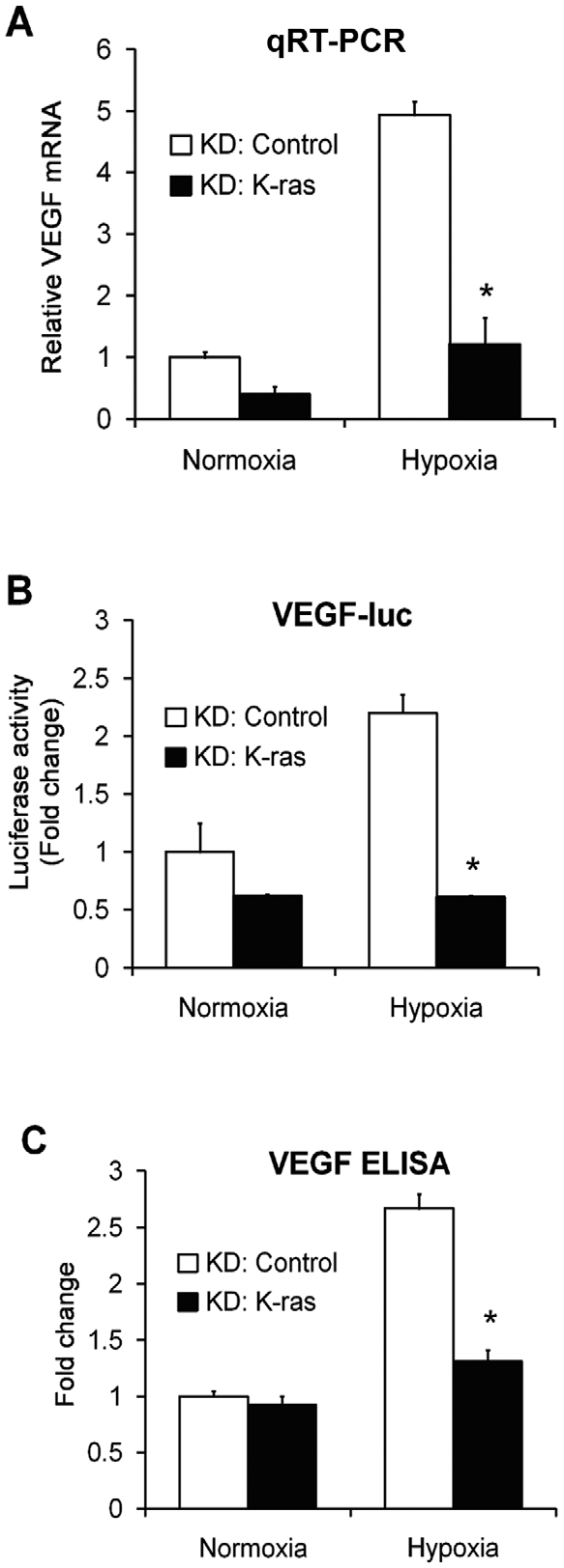
Hypoxic regulation of VEGF by K-ras. **A**, Relative mRNA levels of VEGF, as evaluated by quantitative RT-PCR, in Caco2 cells transfected with a K-ras-specific siRNA construct or a non targeting control and exposed to normoxia or hypoxia. The data are expressed as fold change as compared to siRNA control cells in normoxia, normalized to 1. Columns, average of at least three experiments; bars, SEM. *, P<0.05 as compared to control cells. **B**, Caco2 cells were transiently transfected with either siRNA targeting endogenous K-ras or non targeting control siRNA. After 24 hours, a 2.3 kb VEGF-luciferase reporter construct was co-transfected with pRL-CMV and cells were incubated in normoxia or hypoxia for additional 24 hours. The data are expressed as fold change as compared to siRNA control cells in normoxia, normalized to 1. Columns, average of at least three experiments; bars, SEM. *, P<0.05 as compared to control cells. **C**, Supernatant from cells in (**A**) was collected, and an ELISA for VEGF was performed. The data are expressed as fold change as compared to siRNA control cells in normoxia, normalized to 1. Columns, average of at least three experiments; bars, SEM. *, P<0.05 as compared to control cells.

Hypoxia is also a potent stimulus for angiogenesis through the induction of VEGF. We previously demonstrated that hypoxia and oncogenic K-ras can synergistically up-regulate VEGF [2]. While such studies provide invaluable insights into oncogenic K-ras function, they do not provide insights into mechanisms that colon cancer cells with a wild-type *KRAS* may utilize. We therefore inhibited the endogenous wild-type *KRAS* in Caco2 cells with siRNA oligos to define the relationship between K-ras activation and VEGF production. In Caco2 cells transfected with a control siRNA, hypoxia increased VEGF mRNA levels 4.9-fold. Knockdown of K-ras resulted in a decrease of VEGF mRNA expression by 60% and 76% in normoxic and hypoxic conditions, respectively, when compared to cells transfected with a control siRNA (Fig. 5A). We next used a VEGF promoter reporter construct (Fig. 5B). Hypoxic conditions induced VEGF promoter activity by 2.2 fold. Consistent with our qRT-PCR results, silencing of K-ras dramatically reduced the hypoxic induction of VEGF promoter activity by 72%. In normoxia, there was a 38% reduction in VEGF promoter activity after K-ras silencing. This decrease in VEGF promoter activity correlated with the changes seen in K-ras activation. Finally, an ELISA assay demonstrated that secreted VEGF protein levels were up-regulated 2.6-fold by hypoxia. Knockdown of wild-type *KRAS* in Caco2 cells decreased VEGF protein levels by only 8% in normoxia but reduced VEGF protein levels more significantly in hypoxia by 51% (Fig. 5C). These results suggest that a wild-type *KRAS* gene is also a critical regulator of VEGF in hypoxic conditions.

### Hypoxic induction of VEGF is also Src-dependent

**Figure 6:**
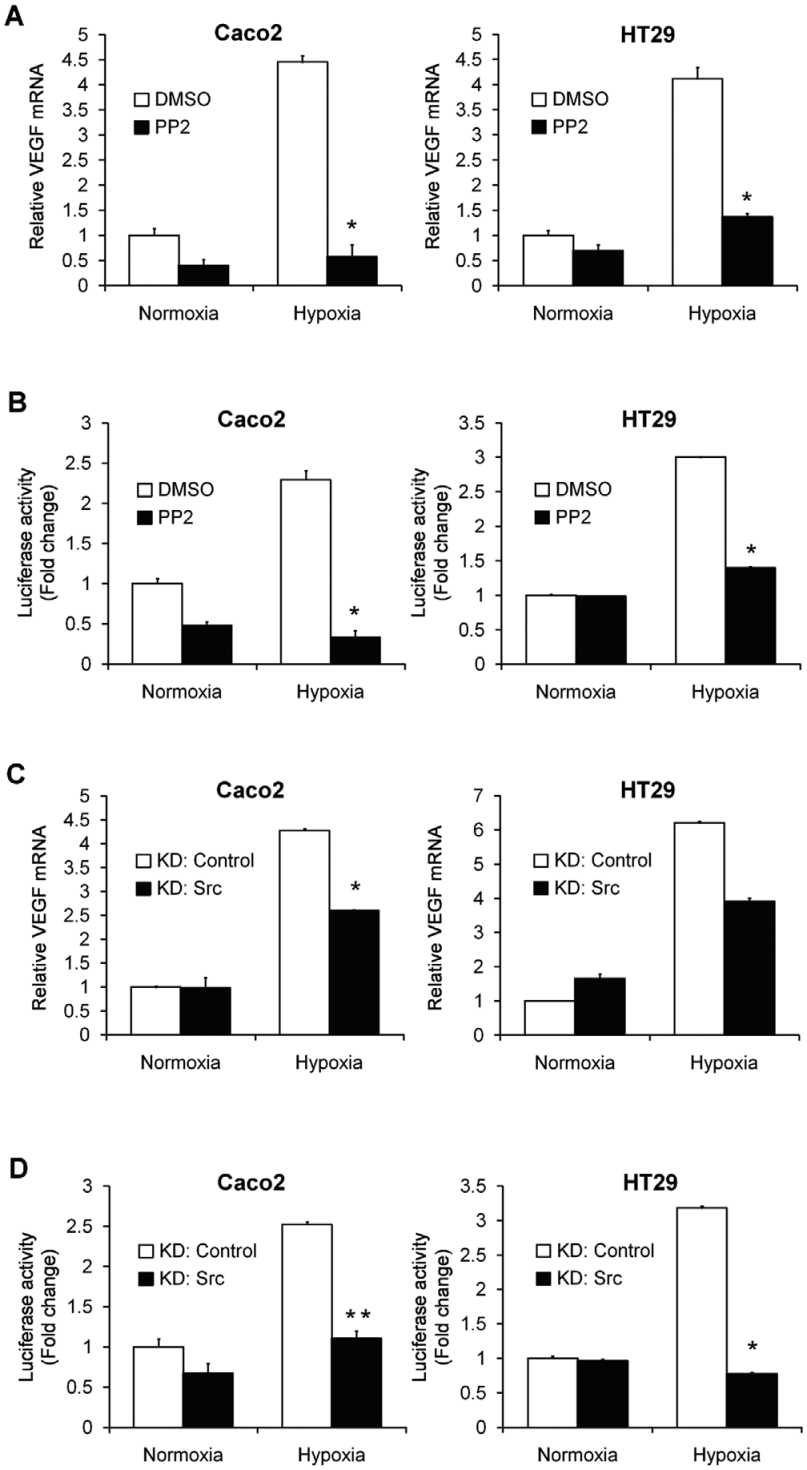
Induction of VEGF under hypoxic conditions is suppressed by inhibition of c-Src. **A and C**, Relative mRNA levels of VEGF, as evaluated by quantitative RT-PCR, in Caco2 and HT29 cells pretreated with 10 µM PP2 (**A**) or transiently transfected with a Src-specific siRNA construct (**C**), and exposed to normoxia or hypoxia for 24 hours. The data are expressed as fold change as compared to control cells in normoxia, normalized to 1. *, P<0.05. **B and D**, VEGF luciferase reporter assays of Caco2 and HT29 cells, pretreated with 10 µM PP2 (**B**) or transiently transfected with a Src-specific siRNA construct (**D**), and exposed to normoxia or hypoxia for 24 hours. The results are from three independent experiments carried out in duplicate and are presented as fold change as compared with control cells in normoxia normalized to 1. Data are shown as mean ± SD. *, P<0.05. **, P<0.01.

Although previous evidence has indicated that c-Src signal transduction pathways can regulate VEGF expression, the relationship between activation of Src by hypoxia and production of VEGF in colon tumor cells has not yet been clarified. Rather than overexpressing v-Src, we blocked endogenous c-Src with PP2. PP2 treatment for 24 hours in Caco2 cells suppressed VEGF mRNA levels by 87% in hypoxia compared to a 60% decrease in normoxia (Fig. 6A). PP2 also suppressed VEGF promoter activity by 85% in hypoxia compared to a 50% reduction in normoxia (Fig. 6B).

To verify the specific role of c-Src in the hypoxic induction of VEGF, we utilized Src siRNA oligos. With this approach, mRNA levels of VEGF were unchanged in normoxic conditions but reduced by 39% in hypoxia (Fig. 6C), and VEGF promoter activity decreased 56% compared with a 33% decrease in normoxia (Fig. 6D). To confirm this effect was not unique to Caco2 cells, HT29 cells were also analyzed. Treatment with PP2 or Src siRNA oligos in HT29 cells under normoxic conditions had negligible effects on VEGF mRNA or promoter activity. In contrast, under hypoxic conditions, PP2 suppressed VEGF mRNA levels and promoter activity by 67% and 53%, respectively (Fig. 6A and 6B, right panels); similarly, c-Src siRNA oligos reduced VEGF mRNA levels and promoter activity by 37% and 76% in hypoxia, respectively (Fig. 6C and 6D, right panels). Collectively, these results also implicate c-Src as a critical regulator of VEGF in hypoxia.

## Discussion

Hypoxia is an unavoidable consequence of rapid tumor growth that outstrips the existing blood supply. A carefully orchestrated set of adaptive responses ensures survival of the tumor cell in these hypoxic conditions. The HIF-1 transcription factor is known to play a role in this hypoxic response [13], [14], [15]. We sought to determine whether additional oncogenic pathways may enhance these adaptive responses. In colon cancer, previous studies have demonstrated a synergistic interaction between *KRAS* mutations and hypoxia in the regulation of multiple genes including VEGF [2], [8], [9]. However, *KRAS* mutations are identified in less than half of all colon cancers, and the role of wild-type *KRAS* in the hypoxic response is less certain. To our knowledge, this is the first demonstration of the hypoxic activation of K-ras in colon cancer.

Our data demonstrate that hypoxia is a potent activator of wild-type K-Ras in colon cancer cells. Activation of K-ras resulted in the downstream activation of Akt, induction of VEGF, and inhibition of apoptosis. These effects on angiogenesis as well as apoptosis are both critical for tumor survival in conditions of sustained hypoxia. The recruitment of the Akt pathway is consistent with previous reports of hypoxic signaling by mutant K-ras in the regulation of OPN gene expression [9]. K-ras can activate hypoxia-inducible factor-1 (HIF-1) through protein phosphorylation, and some of the observed effects on the induction of VEGF may potentially be mediated through HIF-1 [16]. However, it is unlikely that HIF-1 is the only mediator of this process, as K-ras can also regulate VEGF through HIF-1 independent pathways in hypoxia [17].

The activation of K-ras in hypoxia depended upon the upstream activation of c-Src. There is a precedent for the hypoxic activation of Src, which has been demonstrated both *in vivo* and *in vitro* in other cell types [18]. There are a number of specific mediators that link Src to K-ras, and a role for the activation of EGFR is well-described [11], [19], [20]. Despite its established link to colonic tumorigenesis, EGFR does not play a role in the hypoxic activation of K-ras.

Cellular responses to hypoxia seek to preserve survival in a hostile microenvironment. The activation of K-ras in hypoxic conditions implies a key role in the hypoxic response, and our studies highlight two important functions: inhibition of apoptosis and stimulation of angiogenesis. The role of K-ras in the regulation of apoptosis is highly dependent upon context and cell type. In some cases, K-ras can be pro-apoptotic through the activation of RASSF1 or Nore1, but in other scenarios it may serve anti-apoptotic functions through PI3K or Tiam1 [21]. Our studies in colon cancer cells demonstrate a key role for activation of the Akt pathway in hypoxia, which has been previously shown to block apoptosis in other cellular systems [22], [23], [24]. Interestingly, ERK was not activated by hypoxia in our system. The role of ERK activation in colon tumors is not straightforward. Although expression of oncogenic K-ras in normal colonic epithelial cells can strongly activate ERK *in vivo*, only limited ERK signaling was observed in colon tumors that developed in these animals [10]. In addition, colon cancer cells carrying a K-ras mutation failed to demonstrate significant activation of ERK, and no reliable correlation between *KRAS* mutation status and ERK activation has been observed in human colon cancer samples [10], [25]. The specific mechanisms that determine which effector pathways are engaged by K-ras in hypoxic versus normoxic conditions remain to be defined and illustrates the plasticity of K-ras function depending upon specific micro-environmental cues.

Hypoxic conditions can therefore create a milieu in which proto-oncogenes that are not mutated can mimic activated oncogenes. Even though wild-type K-ras can be activated by hypoxia, it is likely that its spectrum of activity does not entirely overlap with that of mutant K-ras and there are other properties specific to oncogenic *KRAS* alleles. Activation of Akt in hypoxia appears to be a common feature of colon tumors with or without *KRAS* mutations, but it has become clear that mutant K-ras and physiologically activated wild-type K-ras do not function identically [26]. Nevertheless, these findings indicate a critical role for wild-type *KRAS* alleles in hypoxia and provide a potential explanation for the aggressive behavior of tumor cells that can survive in the hypoxic microenvironment.

## Materials and Methods

### Reagents

The antibodies used in this study were purchased from the following vendors: p-Akt (Ser^473^), p-MAPK (Thr^202^-Tyr^204^), p-Src (Tyr^416^), p-EGF Receptor (Tyr^1068^), total Akt, MAPK, Src, and EGF receptor, were from Cell Signaling Technology (Beverly, MA); v-Src (Ab-1, clone327) was from Calbiochem (San Diego, CA); K-ras and N-ras were from Santa Cruz Biotechnology (Santa Cruz, CA).

### Cell lines and culture conditions

Human colon cancer cell lines were obtained from the American Type Culture Collection. Cells were maintained in Dulbecco's modified Eagle's medium (DMEM; GIBCO-Invitrogen, Carlsbad, CA) supplemented with 10% fetal bovine serum (FBS; HyClone, Ogden, UT) and antibiotics (Penicillin-Streptomycin, Invitrogen). Hypoxic conditions were achieved by culturing cell lines in a sealed hypoxia chamber (Billups-Rothenberg, Del Mar, CA) with a mixture of 1% O_2_, 5% CO_2_, and 94% N_2_. Cells were switched to serum-free UltraCulture (Lonza, Allendale, NJ) before hypoxia. The Src kinase inhibitor PP2 and antioxidant NAC (Calbiochem) were added 1 hour prior to exposure to normoxia or hypoxia. Acidic DMEM was prepared by dissolving DMEM powder without sodium bicarbonate and sodium pyruvate (Invitrogen) in distilled H_2_O, adjusting the pH to 6.5, filtering and storing at 4°C.

### Real Time PCR Assay

RNA was extracted using the ISOGEN kit (Nippon gene, Tokyo, Japan) and quantitative reverse transcription PCR was performed using the iScript^TM^ cDNA Synthesis kit (BIO-RAD, Hercules, CA). 18S rRNA served as an endogenous control. We used a Power SYBR Green Master Mix (Applied Biosystems, Foster City, CA) and iQ5 Real-time PCR detection system (BIO-RAD) for real-time quantification.

### siRNA analysis

siRNA specific against K-ras (Ambion ID: 6752 and ID: 120702; Austin, TX) and c-Src (on-target plus SMART pool human c-Src, Dharmacon Inc. ID: L-003175-00 and Ambion ID: s13414; Lafayette, CO) were utilized. A control siRNA that does not correspond to any known human gene was also utilized. Cells were plated to reach 30-50% confluence on the day of transfection and 20 nM siRNA duplexes were introduced using Lipofectamine RNAiMAX (Invitrogen).

### Establishment of stable cells

Lentivirus vector pCSGW GFP-LC3 was kindly provided by Dr. Ramnik Xavier (Massachusetts General Hospital, Boston, MA, USA), and the GFP-LC3 fragment was excised with BamHI and NotI. The human K-ras cDNA was amplified by reverse transcription-polymerase chain reaction (RT-PCR) using mRNA from SW480 cells that have homozygous mutation at codon 12 of *KRAS* gene (G12V). pCSGW K-ras V12 or empty vector were introduced with packaging plasmid pCMV-dR8.91 and envelope plasmid pMD2.G into HEK293T cells by transfection with the Fugene6 regent (Roche, Indianapolis, IN). The culture media containing lentivirus was harvested twice every 24 hours and filtered, and polybrene was added at 8 µg/mL. Caco2 cells were infected by culturing with the virus containing medium for 48 hours.

### Transfection

Caco2 cells were transfected with empty vector (pEVX) or constitutively active c-Src plasmid (Src Y527F) using Lipofectamine 2000 (Invitrogen). Forty-eight hours after transfection, cells were harvested for a Ras activation assay. Plasmids containing c-Src gene mutants and pEVX vector were kind gifts from Dr. David Shalloway and Dr. Michael Botchan, respectively (Addgene, Cambridge, MA).

### Reporter assays

Luciferase reporter assays were performed in 24-well tissue culture plates. Cell were cotransfected with 0.6 µg of a 2.3 kb VEGF-luciferase reporter construct and 2 ng of pRL-CMV (Promega, Madison, WI) using Fugene6 (Roche) [17]. Twenty-four hours later the media was switched to UltraCulture and cells were exposed to normoxia or hypoxia for additional 24 hours. Luciferase activity was measured with a dual luciferase reporter assay system (Promega).

### ELISA

Culture media and cellular extracts were collected after 24 hours of normoxic or hypoxic conditions. VEGF protein levels were assayed using a human VEGF-specific ELISA Kit (Quantikine; R&D Systems, Minneapolis, MN) and measured by a microplate reader set to 450 nm.

### Western blotting

Protein lysates were harvested from cells incubated in normoxia or hypoxia for the indicated periods. Cells were lysed in chilled lysis buffer (Cell Signaling) with Protease inhibitor cocktail (Roche). Lysates (18–50 µg) were resolved on a Bis-Tris polyacrylamide or Tris-Acetate gel (Invitrogen) and transferred onto a polyvinylidene fluoride (PDVF) membrane (Millipore, Bedford, MA). Membranes were blocked in 5% non-fat milk containing 0.1% Tween 20 (TBS-T) for 1 hour at room temperature and incubated overnight with relevant antibodies at 4°C. Secondary antibodies coupled to horseradish peroxidise were visualized using the Western Lighting Chemiluminescence Reagent Plus (PerkinElmer Life Sciences, Boston, MA).

### Ras activation assay

The level of active Raf-bound Ras was assessed utilizing a Ras activation assay kit (Upstate, Lake Placid, NY). Cells were cultured to 70%–80% confluence in a 15 cm dish and switched to DMEM with 2% FBS before incubation in normoxia or hypoxia for 4 hours. Two milligrams of each cell extract was mixed with 10 µL of the Ras Assay Reagent (Raf-1 RBD agarose slurry) and incubated at 4°C for 45 minutes. The agarose beads were resuspended in 40 µL of 2x Laemmli reducing sample buffer and boiled. Twenty microliters of lysates were separated by SDS-PAGE and transferred to a PVDF membrane. Active Ras was detected using a Ras antibody (kit component) or an isoform specific antibody to K-ras or N-ras (both Santa Cruz).

### Annexin V-PI staining for fluorescence microscopy

An apoptosis assay was performed using ApoAlert Annexin V kit (Clontech, Palo Alto, CA). Colo320DM cells grown on sterile coverslips were transfected with control or K-ras siRNA, incubated in hypoxia for 48 hours, and then incubated with Annexin V-FITC and Propidium Iodide (PI) in the dark for 10 minutes at room temperature. Coverslips were inverted on glass slides and cells were visualized with an Olympus AX70 microscope (Olympus, Canter Valley, PA).

### Determination of apoptosis by flow cytometry

Caco2 cells were transfected with K-ras or control siRNA 24 hours before incubation in hypoxia. Cells which were irradiated under UV before incubation with Cycloheximide (10 µg/mL) and TNFα (100 ng/mL) in normoxia served as a positive control. Cells were trypsinized and resuspended in 200 µL binding buffer (ApoAlert Annexin V kit) containing 5 µL of annexin V-FITC stock and 10 µL of a 50 µg/mL solution of propidium iodide (PI). After incubation for 15 minutes at room temperature, the samples were analyzed by FACSCalibur (BD Bioscience, Bedford, MA) using Flowjo software. For each measurement, 100,000 cells were collected.

### Statistical analysis

Statistical differences were analyzed by the Student's *t* test, and *P* values<0.05 were considered statistically significant.

## Supporting Information

Figure S1 N-ras is not activated by hypoxia.
(0.15 MB DOC)

Figure S2 Over-expression of c-Src increases K-ras activity.
(0.10 MB DOCX)
