# Supplementary material for: Hypoxia Activates the K-Ras Proto-Oncogene to Stimulate Angiogenesis and Inhibit Apoptosis in Colon Cancer Cells
Source: PLoS One. 2010 Jun 4;5(6):e10966. doi: 10.1371/journal.pone.0010966 (PMC2881039; doi:10.1371/journal.pone.0010966)
Supplement: Figure S1 — N-ras is not activated by hypoxia. (0.15 MB DOC) [file pone.0010966.s001.doc]

**SUPPORTING INFORMATION S1**


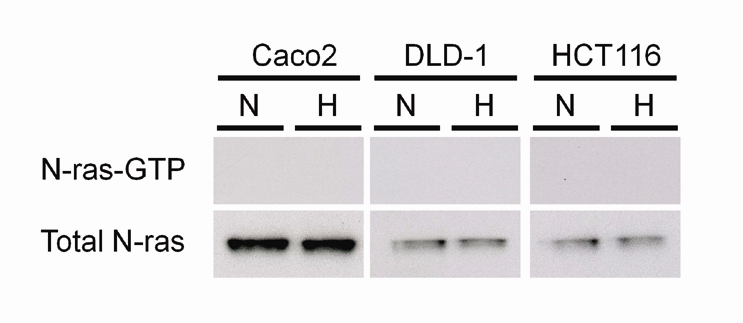


**Figure S1. N-ras is not activated by hypoxia.** Caco2, DLD1, and HCT116 cells were incubated in hypoxia. A Ras activation assay was performed and blots were re-probed with an N-ras specific antibody.
