# Supplementary material for: Hypoxia Activates the K-Ras Proto-Oncogene to Stimulate Angiogenesis and Inhibit Apoptosis in Colon Cancer Cells
Source: PLoS One. 2010 Jun 4;5(6):e10966. doi: 10.1371/journal.pone.0010966 (PMC2881039; doi:10.1371/journal.pone.0010966)
Supplement: Figure S2 — Over-expression of c-Src increases K-ras activity. (0.10 MB DOCX) [file pone.0010966.s002.docx]

**SUPPORTING INFORMATION S2**


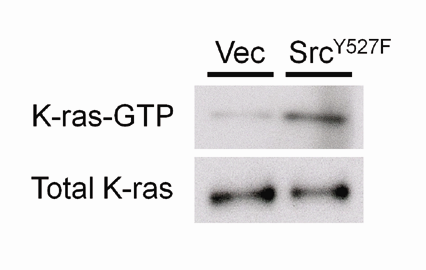


**Figure S2. Over-expression of c-Src increases K-ras activity.** Caco2 cells were transfected with a c-Src expression vector. Activated Ras was pulled down and membranes were re-probed with a specific K-ras antibody.
